# A standardized extract of *Coleus forskohlii* root protects rats from ovariectomy-induced loss of bone mass and strength, and impaired bone material by osteogenic and anti-resorptive mechanisms

**DOI:** 10.3389/fendo.2023.1130003

**Published:** 2023-02-28

**Authors:** Chirag Kulkarni, Shivani Sharma, Konica Porwal, Swati Rajput, Sreyanko Sadhukhan, Vaishnavi Singh, Akanksha Singh, Sanjana Baranwal, Saroj Kumar, Aboli Girme, Alka Raj Pandey, Suriya Pratap Singh, Koneni V. Sashidhara, Navin Kumar, Lal Hingorani, Naibedya Chattopadhyay

**Affiliations:** ^1^ Division of Endocrinology and Centre for Research in Anabolic Skeletal Targets in Health and Illness (ASTHI), Council of Scientific & Industrial Research-Central Drug Research Institute, Lucknow, India; ^2^ Academy of Scientific and Innovative Research (AcSIR), Ghaziabad, India; ^3^ Department of Mechanical Engineering, Indian Institute of Technology Ropar, Rupnagar, Punjab, India; ^4^ Pharmanza Herbal Pvt. Ltd., Anand, Gujarat, India; ^5^ Medicinal and Process Chemistry Division, Council of Scientific & Industrial Research (CSIR)-Central Drug Research Institute, Lucknow, India; ^6^ Sophisticated Analytical Instrument Facility & Research, Council of Scientific & Industrial Research-Central Drug Research Institute, Lucknow, India

**Keywords:** *Coleus forskohlii*, osteotomy, new bone formation, post-menopausal osteoporosis, micro-computed tomography, bone material and quality, histomorphometry

## Abstract

**Introduction:**

In obese humans, *Coleus forskohlii* root extract (CF) protects against weight gain owing to the presence of forskolin, an adenylate cyclase (AC) activator. As AC increases intracellular cyclic adenosine monophosphate (cAMP) levels in osteoblasts that has an osteogenic effect, we thus tested the skeletal effects of a standardized CF (CFE) in rats.

**Methods:**

Concentrations of forskolin and isoforskolin were measured in CFE by HPLC. CFE and forskolin (the most abundant compound present in CFE) were studied for their osteogenic efficacy *in vitro* by alkaline phosphatase (ALP), cAMP and cyclic guanosine monophosphate (cGMP) assays. Femur osteotomy model was used to determine the osteogenic dose of CFE. In growing rats, CFE was tested for its osteogenic effect in intact bone. In adult ovariectomized (OVX) rats, we assessed the effect of CFE on bone mass, strength and material. The effect of forskolin was assessed *in vivo* by measuring the expression of osteogenic genes in the calvarium of rat pups.

**Results:**

Forskolin content in CFE was 20.969%. CFE increased osteoblast differentiation and intracellular cAMP and cGMP levels in rat calvarial osteoblasts. At 25 mg/kg (half of human equivalent dose), CFE significantly enhanced calcein deposition at the osteotomy site. In growing rats, CFE promoted modeling-directed bone formation. In OVX rats, CFE maintained bone mass and microarchitecture to the level of sham-operated rats. Moreover, surface-referent bone formation in CFE treated rats was significantly increased over the OVX group and was comparable with the sham group. CFE also increased the pro-collagen type-I N-terminal propeptide: cross-linked C-telopeptide of type-I collagen (PINP : CTX-1) ratio over the OVX rats, and maintained it to the sham level. CFE treatment decreased the OVX-induced increases in the carbonate-to-phosphate, and carbonate-to-amide-I ratios. CFE also prevented the OVX-mediated decrease in mineral crystallinity. Nanoindentation parameters, including modulus and hardness, were decreased by OVX but CFE maintained these to the sham levels. Forskolin stimulated ALP, cAMP and cGMP *in vitro* and upregulated osteogenic genes *in vivo*.

**Conclusion:**

CFE, likely due to the presence of forskolin displayed a bone-conserving effect *via* osteogenic and anti-resorptive mechanisms resulting in the maintenance of bone mass, microarchitecture, material, and strength.

## Introduction

*Coleus forskohlii* (CF), commonly known as Coleus in English, is a medicinal herb with rich ethnopharmacological applications. CF is also used in Ayurvedic medicine to treat a variety of ailments, including inflammatory diseases, hypertension, respiratory disorders, aging, and weight management (1, 2). CF is a rich source of secondary metabolites, including terpenoids, flavonoids, and alkaloids (3). The major bioactive compound of Coleus root is forskolin, a labdane diterpene which is of clinical interest because of its weight-loss property. CF is the only species known to contain significant amount of forskolin (4). Forskolin acts by increasing the accumulation of cyclic adenosine monophosphate (cAMP) without hormonal stimulation of adenylate cyclase (AC) (2, 5). cAMP binds to, and activates protein kinase A (PKA), which then activates lipases by phosphorylating them, resulting in lipolysis. Given this mechanism of action of forskolin, CF extract is thought to have an anti-obesity effect that has been demonstrated in a few preclinical studies (5–8). Human studies, albeit scanty present inconsistent outcomes in reducing the weight of obese men and women (5, 9, 10) although it could attenuate weight gain (10). Despite lack of strong evidence base from human studies concerning its weight-reduction effect, CF standardized to contain 10-20% forskolin is widely available as a dietary supplement.

Activation of AC by forskolin resulting in the rise in intracellular cAMP appears to underlie the osteogenic effect of the compound. The osteogenic drugs teriparatide (PTH 1-34) and abaloparatide (PTHrP 1-36) act by type 1 PTH receptor to activate AC to increase intracellular cAMP in osteoblasts (11). However, cAMP can either stimulate or inhibit osteogenic differentiation in human mesenchymal stem cells, depending on the duration of the rise in its intracellular levels (12). Rises in the intracellular cAMP stimulate mesenchymal stem cells (MSC) proliferation and differentiation to osteoblasts, as well as osteoblastic differentiation from pre-osteoblasts (13). db-cAMP, a synthetic cAMP analog, stimulated osteogenic differentiation *in vitro* and new bone formation *in vivo* (14). Sustained stimulation of cAMP signaling, on the other hand, decreases osteoblast differentiation and mineralization (15) while inducing adipocyte differentiation (13). We previously observed that the profile of cAMP activation kinetics of forskolin matches with PTH (15), which led us to surmise that CF rich in forskolin may have an osteogenic property.

Here, we used a standardized preparation of CF root extract (CFE, rich in forskolin) and studied the osteogenic and anti-osteoporotic effects in rats. Three models were used for these purposes, i) femur osteotomy model (for rapid assessment of bone regeneration) for determining the osteogenic dose of CFE, ii) growing rats for determining the modeling-directed bone formation in intact rat bones, and iii) OVX rats (a model for postmenopausal osteopenia) to assess the effect of CFE on maintaining bone mass, microarchitecture, bone formation, bone turnover, bone strength, and bone quality. *Ex vivo* cultures of bone marrow cells were used to evaluate the effect of CFE on osteoblast differentiation. Finally, we determined the amount of forskolin in CFE and assessed its *in vivo* osteogenic effect.

## Materials and methods

### Plant material, chemicals, and reagents

CFE used in this study was procured from Pharmanza Herbal Pvt. Ltd (Anand, Gujrat, India). Forskolin was procured from Phytocompounds (Bangalore, India). Acetonitrile and methanol MS grade were procured from JT Baker and Rankem. Cell culture medium and all chemicals were procured from Sigma-Aldrich (St. Louis, MO, USA). FBS, collagenase and diaspase were purchased from Invitrogen (Carlsbad, CA, USA). Gum acacia was purchased from Santa Cruz Biotechnology, Inc. (Dallas, TX, USA).

### Preparation of analytical solutions for high-performance liquid chromatographic-based study

The solution of forskolin standard 1.0 mg/mL (1000 µg/ml) was prepared by dissolving 10.0 mg forskolin in 5.00 ml acetonitrile and making up the volume up to 10.0 ml in acetonitrile. The sample for CFE (1 mg/ml) was prepared by dissolving 25.0 mg sample in 15.0 ml of acetonitrile and making up the volume 25.0 ml. The mixture was then centrifuged, filtered, and used for further analysis using HPLC according to our previously described method (16).

### Chromatographic conditions

The analysis was performed on a Phenomenex^®^ Luna C18 column (250 ×4.6 mm, 5 μm). With gradient elution of water and acetonitrile at a flow rate of 1.8 ml/min was carried out as follows: 0.01 – 25 min, 45% B; 25 – 28 min, 45-95% B; 28 – 35 min, 95% B; 35 –36 min, 95-45% B; 36 - 45 min, 45% B at 220 nm wavelength for UV. The column temperature was kept at 30°C, with an injection volume was 20.0 μL (16).

### *In vivo* studies

### Animal studies

#### Ethics statement and husbandry

Animal husbandry and all animal experimental procedures were prior approved by the Institutional Animal Ethics Committee (Registration no.: 34/GO/ReBiBt-S/Re-L/99 CPCSEA) (IAEC/2021/16/Renew-0/Dated-04/01/2021). Female Sprague Dawley (SD) rats were obtained from the National Laboratory Animal Centre, CSIR-CDRI, Lucknow, and kept under controlled conditions: temperature (22-25°C), humidity (50-60%), and a 12-hour light/dark cycle. The rats were acclimated for 8 days prior to surgery. The rats were maintained on standard rodent chow diet and purified water ad libitum during the experimental period. Rats received intramuscular ketamine (40 mg/kg) and xylazine (10 mg/kg) anaesthesia prior to all surgeries.

#### Femur osteotomy model

Adult rats with a drill-hole (0.8mm) osteotomy in the femur diaphysis provide a rapid and reliable model for evaluating bone regeneration that is proportional to bone formation (17, 18). Furthermore, this model is useful for the determination of effective osteogenic dose of a drug.

Twenty four female SD rats (220 ± 20 g) were used for femur osteotomy following a previously described protocol (19). Post-surgery, rats were randomly divided into four groups (n=6 rats/group); vehicle (water, orally), CFE (25-, 50- and 100 mg/kg, orally). All the treatments were given daily for 12 days. 24 h before sacrifice, all the animals were given subcutaneous (s.c.) injections of calcein (20 mg/kg). After sacrifice, bones were collected and processed for calcein labeling studies according to our previously published protocol. 60 μm sections were made through the osteotomy site using Isomet-Slow Speed Bone Cutter (Buehler, Lake Bluff, IL, USA) (19, 20). Sections were photographed using a confocal microscope (Leica TCS SP-8, Wetzlar, Germany) and analyzed using LAS-X software.

#### Modeling-directed bone formation model

Modeling-directed bone formation is the dominant event in growing rats, which enables evaluation of the osteogenic response in intact skeleton (21, 22). Accordingly, 1-month-old 12 SD female rats (65 ± 5 g) were randomly divided into two groups (n=6/group): vehicle (water), CFE (25 mg/kg; oral). Treatments were given for 1 month. For dynamic histomorphometry study (to measure surface-referent bone formation parameters), each animal was given two s.c. injections of calcein (20 mg/kg) at 10 days interval before sacrifice. At the end of the experiment, bones were collected for μCT, bone strength, and histomorphometry analyses, for which the details are given below. Femur length was measured with a Vernier caliper.

#### Post-menopausal osteoporosis model

Adult rats with bilateral OVX is a widely used model for post-menopausal osteoporosis, which, we used to evaluate CFE’s prophylactic anti-osteoporosis impact. To this aim, 24 SD rats (220 ± 20 g, 3 months old) underwent either bilateral OVX or a sham operation (ovary intact) (19, 23). Following surgery, the OVX rats were randomized into two equal groups (n = 8 rats/group) in the presence of two researchers: OVX + vehicle (water); OVX + CFE (25 mg/kg, oral); and the sham-operated animals received vehicle. All the therapies were given daily for 3 months. For dynamic histomorphometry, all animals received 2 doses of calcein (20 mg/kg, s.c.) before sacrifice at the interval of 10 days (19, 20).

After the treatment, serum samples, and bones (femur, tibia, and L5 vertebrae) were dissected and stored at -80°C for further studies.

### Techniques and experimental protocols

#### Body composition analysis

Throughout the experimental period, the body weights of each study group were measured once a week. The body composition of live rats was evaluated by the EchoMRI™-500 body composition analyzer (EchoMRI Corporation Pvt. Ltd. Singapore) 24 h before the end of the experiment (23). Total body mass and lean mass were plotted, while fat mass was plotted by normalizing with the total body mass.

#### μCT analysis of bones

Bone samples were scanned using a SkyScan 1276 computed tomography (μCT) scanner (SkyScan, Ltd., Kartuizersweg, Kontich, Belgium), in accordance with the instructions in our previously published technique (19, 23). CTAn software was used manually to quantify various bone parameters as described previously (24). Reconstructed μCT images underwent a blind evaluation by a third person to determine the extent of bone loss. The degree of bone loss was assessed using reconstructed μCT images that had undergone a blind examination by a third person.

#### L5 compression

Biomechanical strength was measured by L5 compression test using a bone strength tester, TK 252C (Muromachi Kikai Co. Ltd. Tokyo, Japan) according to our previously published method (25).

#### Nanoindentation

The rat femur bone was cut from mid-diaphysis with a low-speed diamond blade saw (IsoMet; Buehler, Lake Bluff, IL, USA), and after that, the samples were kept in epoxy for nearly 2 h for proper cured and, further samples were polished under the ground (Buehler Eco 250 grinder and polisher) with the abrasive papers of 1200, 2000, and 4000 grit size under the cooling condition and polished with a diamond solution of particle sizes of 1, 0.5, and 0.25 µm. After completion of polishing, samples were sonicated for 10 min. The experiment was performed on the T1-950 Tribo Indenter (Hysitron Inc., MN, USA) with Berkovich pyramidal tip in the moist state. Eight indents with a peak load of 3000 µN were applied to cross-section of the bone. The load sequence consists a loading time of 10 s, an unloading segment, and a hold for 10 s. The resultant load-displacement curve was used to calculate the reduced modulus (Er) and hardness (H) by the method of Oliver and Pharr (26, 27).

#### Assessment of bone material

The mineral and collagen properties were analyzed by a Bruker IFS 66v/S Fourier Transformed Infrared (FTIR) spectrophotometer in the attenuated total reflectance mode, under the constant pressure, in the range of 4000 to 400 cm^-1^. From the obtained data, we calculated the following parameters: carbonate-to-phosphate ratio (area ratio of the carbonate peak [852-890 cm^-1^] to phosphate peak [916-1180 cm^-1^]), carbonate-to-amide I ratio (area ratio of the carbonate peak [852-890 cm^-1^] to the amide-1 peak [1596-1712 cm^-1^]) and mineral crystallinity ratio (intensity ratio of [1030 to 1020 cm^-1^]), which is related to crystal size and stoichiometric perfection. The amide I band peak contains several sub-peaks that provide information about the collagen matrix and the location of cross-linkage and non-cross linkage. The sub-band of the amide I peak were fited with Gaussian curves at 1610, 1630, 1645, 1660, 1675, and 1690 cm^-1^ using peak analyzing tools OriginPro 8.5 software (26, 28).

### Bone histomorphometry

Surface-referent bone formation was measured by bone histomorphometry by double calcein labeling in accordance with our previously published protocols to determine the mineralizing surface per bone surface (MS/BS), mineral apposition rate (MAR), and bone formation rate per bone surface (BFR/BS) (19, 20, 29).

#### Measurement of serum bone turnover markers

Rat cross-linked C-telopeptide of type I collagen (CTX-1) kit (Cat. No. E-EL-R1456) and pro-collagen type I N-terminal propeptide (PINP) kit (Cat. No. E-EL-R1414) were purchased from Elabscience, USA, and measured in accordance with manufacturer’s instructions.

#### Measurement of osteogenic gene expression

Rat pups (1 to 2-day-old) were treated with vehicle or forskolin (1- and 2.5 mg/kg) for 5 days. After treatment, calvaria were removed and processed for RNA isolation by trizol method (30). qPCR was performed by SYBR green chemistry (Thermo Fisher Scientific, Ealtham, MA, USA) for the quantitative determination of bone morphogenetic protein 2 (BMP2), type 1 collagen (Col I), receptor activator of nuclear factor kappa-B ligand (RANKL), and osteoprotegerin (OPG) as described previously (31). cDNA was synthesized by using 2μg RNA (Cat no. 4368814, High Capacity cDNA Reverse Transcription Kit, Applied Biosystems by Thermo Fisher Scientific). All genes were analyzed using a real-time PCR machine (QuantStudio^©^ 3 Real-time PCR Instrument, A28132), keeping GAPDH as control. Primer sequences are listed below:

BMP2: Forward 5′-CCCTATATGCTCGACCTGTACC-3′Reverse 5′-GGAAGCTGAGCACGGTGT-3′
Col I: Forward 5′-CCCAGCGGTGGTTATGACT-3′, Reverse 5′- ATCATCGGCCCGGTAGTAA-3′
RANKL: Forward 5′-AGACACAGAAGCACTACCTGA-3′, Reverse 5′-GGCCCCACAATGTGTTGTA-3′;
OPG: Forward 5′-GGAGCTCGAATTCTGCTTGA-3′, Reverse 5′-GAAGAACCCATCCGGACATC-3′;
GAPDH: Forward 5′-TGGGAAGCTGGTCATCAAC-3′, Reverse 5′-GCATCACCCCATTTGATGTT-3′.

#### *Ex vivo* mineralization assay

Bone marrow was collected from rat femur by flushing out using PBS and cells were measured by a hemocytometer. In a 6-well plate, bone marrow cells were seeded at a density of 2 × 10^6^ per well in a differentiating medium (α-MEM with 10 mM β-glycerophosphate, 50 μg/ml ascorbic acid, and 100 nM dexamethasone). After every 48 h media was changed for 21 days. After 21 days, cultures were fixed using 10% formalin and 40 mM Alizarin red-S stain was used to visualize mineralized nodules. 10% cetylpyridinium chloride (CPC) was used to extract the stain and the mineralization was calorimetrically measured at OD 595 nm (32).

### *In vitro* studies

#### Osteoblast culture and ALP assay

Rat pups (1 to 2-day-old) were used to culture calvarial osteoblasts (RCO) as described previously (20). For ALP assay, cells were trypsinized at 90% confluency and seeded in 96-well plate. The adherent cells were treated with CFE (7.8-, 15.63-, 31.25-, 62.5-, 125- and 250 µg/ml) or forskolin (100 pM, 1 nM, 10 nM, and 100 nM) for 48 h in a differentiation medium (α-MEM supplemented with 10 mM β-glycerophosphate and 50 μg/mL ascorbic acid). After 48 h, ALP activity was assessed by adding diethanol amine buffer (DAE) with 2 mg/ml para-nitrophenyl phosphate (pNPP) and measured colorimetrically at OD 405 nm.

#### cAMP and cGMP assays

RCO were treated with CFE or forskolin for 0 min, 5 min, 15 min, 30 min, 60 min and 90 min. After treatments, cAMP and cGMP levels were determined by ELISA kits (Cayman Co., Ann Arbor, MI, USA) in accordance with the manufacturer’s protocol.

#### Statistical analyses

Data are presented as the mean ± standard error of the mean (SEM). One-way ANOVA with a *post hoc* Tukey test using GraphPad Prism 5 and a significance level of 0.05% (95% significance) was used to assess statistical differences between the various treatment groups. An unpaired t-test using GraphPad Prism 5 with a significance level of 0.05% (95% significance) was used in the experiments with two groups to assess statistical differences.

## Results

### Qualitative and quantitative analysis of CFE

The HPLC method was applied for simultaneous quantification of analytes in CFE. The chromatograms for the standard mixture and samples are presented in **Supplementary Figure 1**. The chromatogram of the sample solution obtained in the test for the content of forskolin showed a major peak at a retention time corresponding to that of the forskolin reference standard. Other diterpene peaks in the sample chromatogram exhibited an additional peak corresponding to isoforskolin. The detection was based on approximate relative retention time/minute, which for isoforskolin and forskolin were respectively 0.51 and 1.00 (16). Forskolin and isoforskolin content in the CFE were 20.969% and 3.396%, respectively.

### *In vitro* effect of CFE in RCO

**Figure 1:**
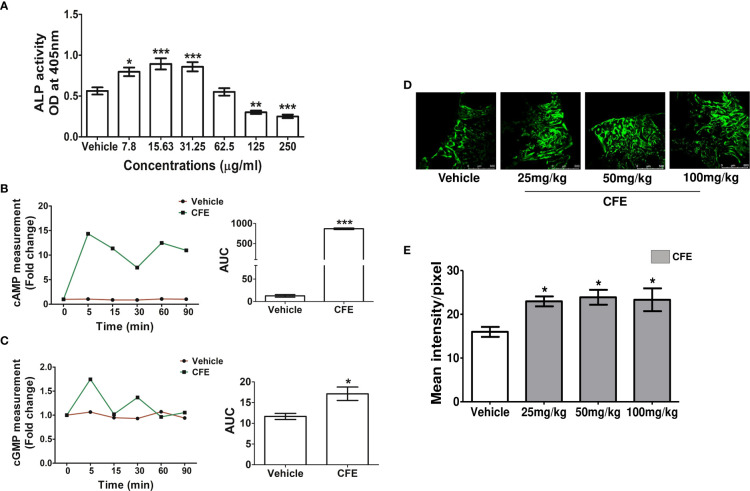
CFE stimulated osteoblast differentiation, cAMP and cGMP *in vitro* and promoted bone regeneration at the femur osteotomy site. **(A)** RCO were treated with CFE, and differentiation was assessed by ALP assay. **(B)** RCO were treated with CFE at the indicated time points and cAMP and **(C)** cGMP production were measured. **(D)** Adult female rats were treated with vehicle and CFE at indicated doses after femur osteotomy for 12 days, and representative images (10X) of calcein deposition at the osteotomy site are shown. **(E)** Quantification of the calcein deposition data are presented (n = 6 bones/group). All data are expressed as mean ± SEM; *p < 0.05, **p < 0.01, ***p<0.001 vs. vehicle.

Osteoblasts are the principal cells that are involved in fracture healing, so we firstly studied the effect of CFE on the differentiation of RCO by ALP activity. At 7.8-, 15.63- and 31.25 μg/ml concentrations CFE significantly increased ALP activity in RCO over the vehicle-treated RCO (**Figure 1A**). As forskolin is an AC activator, we next studied the effect of CFE (15.63 μg/ml) on intracellular cAMP kinetics in RCO and observed a significant increase in the cAMP levels compared with vehicle-treated RCO (**Figure 1B**). Furthermore, at the same concentration, CFE (15.63 μg/ml) increased the cGMP levels compared with the vehicle-treated RCO (**Figure 1C**).

### CFE increased bone regeneration at the fracture site

In obese men, 250mg CFE has been used to study its impact on body mass (5, 9). When converted based on body surface area, rat dose comes to 50 mg/kg. We tested the bone regenerative effect of CFE at 25-, 50- and 100 mg/kg doses by calcein labeling at the femur osteotomy site. Compared with vehicle-treated rats, CFE at all doses significantly increased calcein intensity (**Figures 1D, E**). Since 25 mg/kg dose, which is a half of the human equivalent dose of CFE showed significant bone regenerative effect, we selected this as the minimum effective dose in subsequent studies.

### CFE promoted modeling-based bone growth in female rats

**Figure 2:**
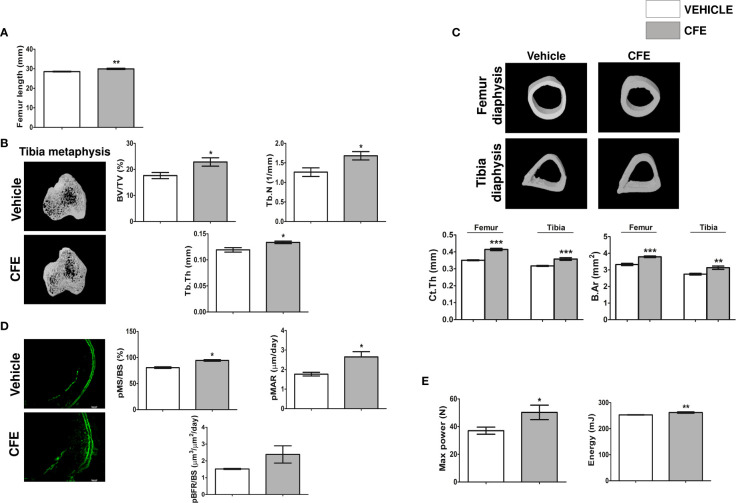
CFE promoted new bone formation in growing rats. **(A)** Femur length. **(B)** Representative μCT images (left panel) and quantitative μCT parameters of the tibia metaphysis (right panel). %BV/TV, percent bone volume per tissue volume; Tb.N, trabecular number; Tb.Th, trabecular thickness. **(C)** Representative μCT images (upper panel) and quantitative μCT parameters of the femur and tibia diaphysis (lower panel). Ct.Th, Cortical thickness and B.Ar, bone area. **(D)** Left panel showing the representative images of double calcein labeling (scale bar, 100 µm) and histomorphometry parameters (right panel) of the indicated groups. **(E)** 3-point bending strength of femur was determined by a bone-strength tester. All data are expressed as mean ± SEM (n = 6 bones/group); *p <0.05, **p <0.01, and ***p<0.001 vs. vehicle-treated group.

Daily supplementation of CFE (25 mg/kg) increased the femur length compared with the vehicle-treated (control) group (**Figure 2A**). Trabecular bones at metaphysis and cortical bone parameters were studied using μCT. Compared to the control group, CFE increased bone volume/tissue volume (BV/TV%), trabecular number (Tb.N) and trabecular thickness (Tb.Th) compared with control (**Figure 2B**). Cortical parameters including cortical thickness (Ct.Th) and bone area (B.Ar) were significantly increased by CFE over the control (**Figure 2C**).

The effect of CFE on bone accrual was measured by dynamic histology by time-spaced calcein labeling study in the periosteal (p) region of the femur diaphysis. Surface-referent bone formation parameters calculated from this study, including pMS/BS (percentage of bone surface undergoing active formation), pMAR (indicating an average rate of osteoblast activity) and pBFR (total bone formation rate during the study period) were significantly increased in the CFE group compared with the control (**Figure 2D**).

Increase in the surface referent bone formation parameters indicative of increased periosteal apposition complemented our observation of higher Ct.Th in the CFE group over the control and is likely to afford greater resistance to fracture (33). Accordingly, we measured the bending strength of femur and observed that maximum power and energy to failure were significantly higher in the CFE group compared with control (**Figure 2E**).

The treatment of CFE had no effect on the body weight compared to the vehicle treated group (data not shown).

### CFE showed bone conserving and osteogenic effect in OVX rats

Because CFE promoted bone regeneration and stimulated modeling-based bone growth, we speculated that it would have bone conserving effect in OVX model of osteopenia. At the end of 3 months of treatment, body composition of all groups were assessed by Echo-MRI. Compared with the sham, OVX rats had increased total body mass, lean mass, and fat mass. CFE had no effect on OVX-induced increase in total body mass and lean mass but significantly decreased fat mass (**Supplementary Figure 2**).

**Figure 3:**
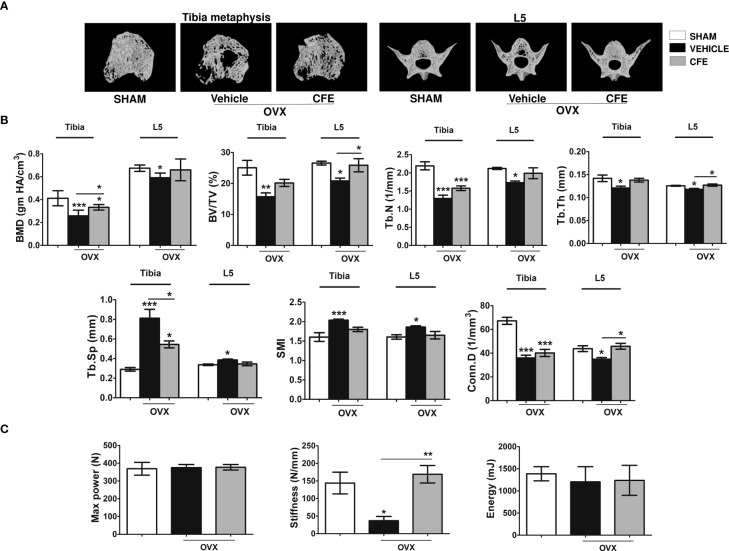
CFE prevented bone loss in osteopenic rats. **(A)** Representative images of tibia metaphysis, and L5 vertebrae are shown. **(B)** Shown are the quantitative μCT parameters of the tibia metaphyses, and L5. BMD, bone mineral density; Tb.Sp, trabecular spacing; Conn.D., connectivity density; and SMI, structure model index. **(C)** The L5 compression strength was determined by a bone-strength tester. All data are expressed as mean ± SEM (n = 6 bones/group); *p <0.05, **p <0.01, and ***p<0.001 vs. sham.

We next studied the effects of CFE on appendicular (tibia) and axial (L5) skeletons of OVX rats (**Figure 3A** for representative images). Bone mineral density (BMD) and BV/TV were significantly decreased in OVX rats compared with sham, and CFE significantly increased these parameters over the OVX. Tb.N and Tb.Th were reduced in the OVX group and CFE significantly increased Tb.Th only. Consequently, trabecular spacing (Tb.sp) that was increased in the OVX group was significantly reduced by CFE treatment. Similar effects were observed in L5 as OVX-induced decrease in BV/TV, Tb.N and Tb.Th were significantly reversed by CFE with consequent recovery of Tb.sp. (**Figure 3B**).

Although there was not a significant difference among groups in maximum power and failure energy of the L5 after the end of the treatment, stiffness was significantly lower in the OVX compared with the other groups (**Figure 3C**).

**Figure 4:**
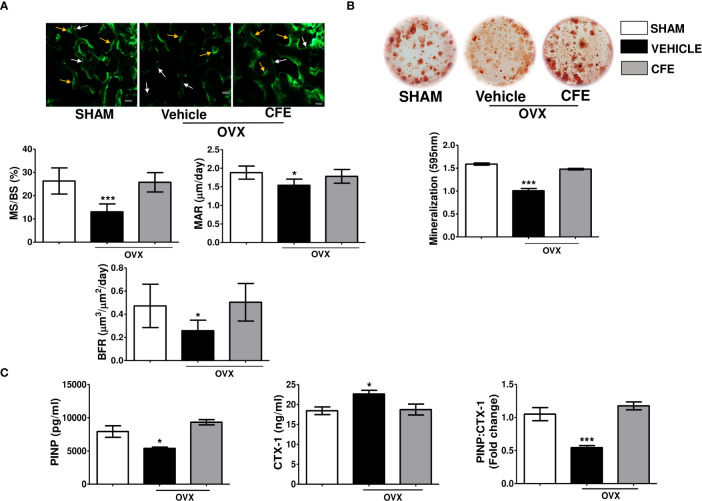
CFE has an osteoanabolic effect in osteopenic rats. **(A)** Upper panel showing representative images (scale bar, 100 µm) of single and double calcein labeled bone surfaces at tibia metaphysis; white arrows- single label and yellow arrows- double label surfaces; and the lower panel showing the histomorphometry parameters in the indicated groups. **(B)** *Ex vivo* mineralization assay was performed in bone marrow stromal cells obtained from the indicated groups. **(C)** Serum procollagen type I N-propeptide (PINP), cross-linked C-telopeptide of type I collagen (CTX1) levels and their ratio were determined by ELISA from the serum of rats with indicated treatments. For ELISA, serum samples of n = 6 rats from each group were taken. For *ex vivo* mineralization femurs and for histomorphometry tibia sections of n = 3 rats from each group were used. All data are expressed as mean ± SEM; *p <0.05, and ***p<0.001 vs. sham.

We next studied whether the preservation of bone mass and strength by CFE in OVX rats occur by an osteogenic mechanism. Dynamic histology of proximal tibia showed significant decreases in MS/BS, MAR and BFR/BS in the OVX rats compared with the sham, and the CFE group completely maintained these parameters to the level of the sham (**Figure 4A**). Complementing the osteogenic effect of CFE through increase in surface-referent bone formation, *ex vivo* mineralization of BM stromal cells in the CFE-treated OVX rats was significantly higher than the OVX group (**Figure 4B**).

Consistent with the *in vivo* and *ex vivo* osteogenic effect of CFE, we observed that the serum bone formation marker, PINP, that was significantly decreased in the OVX group was maintained to the sham level by CFE. Conversely, CFE suppressed the OVX-induced increase in CTX-1, the serum resorption marker. Accordingly, PINP-to-CTX-1 ratio, an indicator of “anabolic window” that was decreased in the OVX group, was maintained to the sham level by CFE (**Figure 4C**).

### CFE improved mineral composition and material properties of bones in OVX rats

The mineral-based parameters including mineral crystallinity and carbonate:phosphate ratio were respectively decreased and increased in the OVX group, and CFE treatment maintained these parameters to the levels of sham. The carbonate:amide-I ratio was significantly increased in the OVX group, while CFE treatment maintain this parameter to the sham level (**Table 1**).

**Table 1:** Bone material and nanoindentation parameters.

Parameters	SHAM	OVX+VEHICLE	OVX+CFE
FTIR			
**Carbonate : Phosphate**	0.0115 ± 0.0007726	0.03041 ± 0.003989***	0.01401 ± 0.0003478
**Mineral crystallinity**	0.9993 ± 0.01559	0.9949 ± 0.001087*	0.9961 ± 0.00054
**Carbonate : Amide I**	0.03869 ± 0.001656	0.08877 ± 0.01514**	0.03525 ± 0.001264
Nanoindentation			
**Modulus (Gpa)**	22.74 ± 1.422	17.23 ± 1.251*	18.74 ± 1.044
**Hardness (Gpa)**	0.9991 ± 0.1281	0.5988 ± 0.07142*	1.146 ± 0.08074

Data are presented as the mean ± SEM (n = 6 rats per group); *p <.05, **p <.01, and ***p<0.001 vs. sham.

We next studied the material properties of bones by nanoindentation. Under a 3000 µN load, the OVX group had significantly lower modulus and hardness compared with sham and CFE groups (**Table 1**).

### Osteogenic effect of forskolin

**Figure 5:**
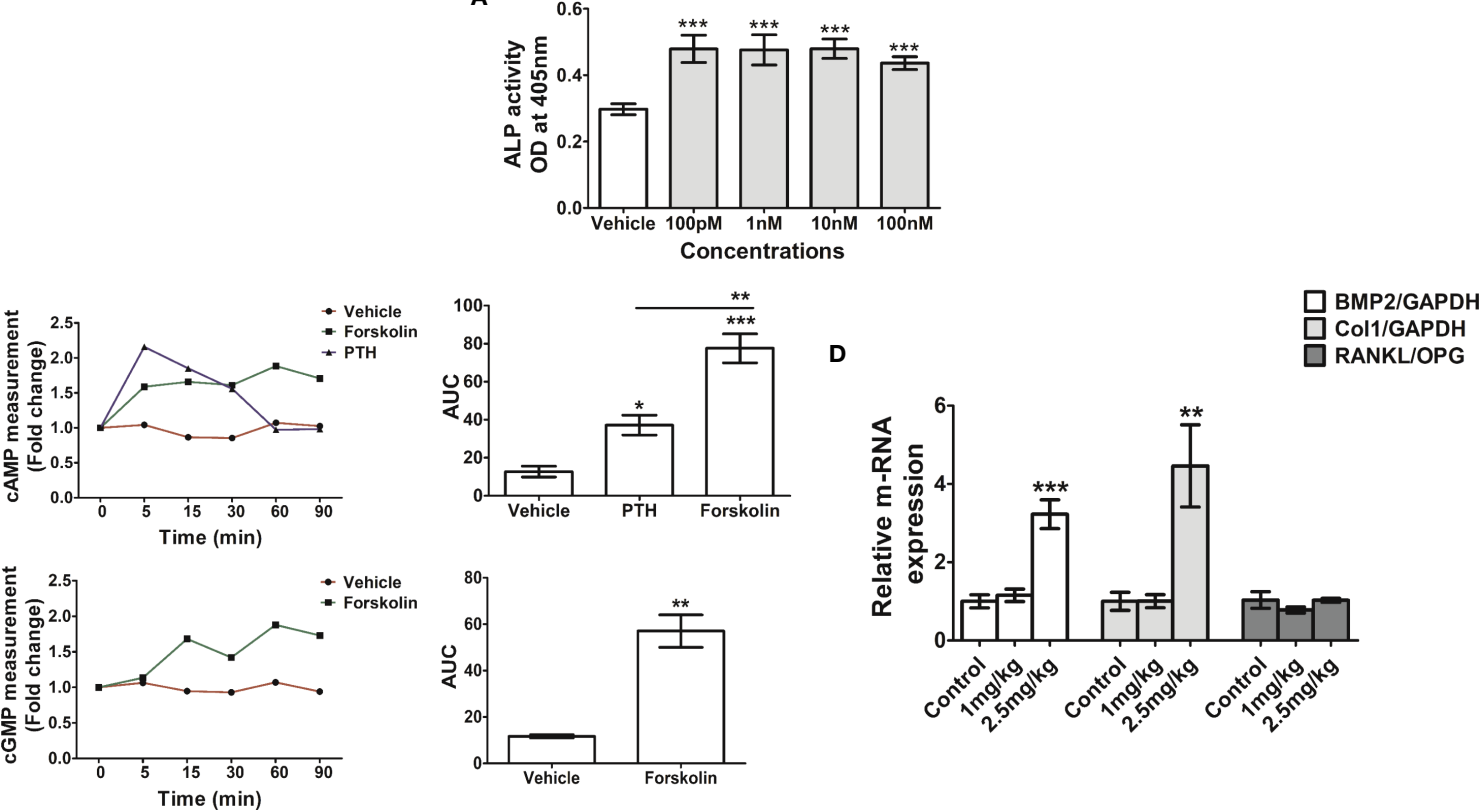
Forskolin has osteogenic effect *in vitro* and *in vivo*. **(A)** RCO were treated with forskolin at the indicated concentrations and differentiation was assessed by ALP assay. **(B)** RCO were treated with forskolin (10nM) for the indicated time points and intracellular cAMP and **(C)** cGMP production were measured. **(D)** Rat pups (1-day old) were injected with forskolin at the indicated doses for 5 consecutive days and the relative expression of osteogenic genes in the calvarial tissue were measured. All values are expressed as mean ± SEM; *p <0.05, **p<0.01 and ***p<0.001 vs. sham.

Since CFE is rich in forskolin we surmised that it contributes to the osteogenic effect of the extract. In the osteoblast ALP assay for assessing differentiation, the EC_50_ of forskolin was 3.8 μM (**Figure 5A**). Prolonged increase in cAMP as caused by theophylline caused osteoblast apoptosis (15). On the other hand, PTH through Gs_α_-coupled activation of PTH receptor-1 stimulates AC to increase osteoblastic cAMP which leads to osteoblast differentiation (34). Therefore, we compared the intracellular cAMP kinetics of forskolin (at 10 nM) with PTH. Forskolin had a greater total cAMP level than PTH (**Figure 5B**). Furthermore, forskolin increased the intracellular cGMP levels in the RCO compared with vehicle treated RCO (**Figure 5C**).

### Forskolin stimulated osteogenic genes' expression *in vivo*

The *in vivo* osteogenic efficacy of forskolin (1- and 2.5 mg/kg) was assessed by injecting it to rat pups and at both the doses forskolin showed positive osteogenic effects. Real-time PCR (qPCR) data showed that forskolin increased the expression of BMP2 and Col I in the treatment groups compared with the vehicle treated pups. There was no change in RANKL/OPG ratio among the groups (**Figure 5D**).

## Discussion

We observed that CFE has osteogenic effect that resulted in a) enhanced bone accrual during growth and b) preservation of bone mass in estrogen deficiency (OVX model). The increase in bone mass by the osteogenic impact of CFE in OVX rats was accompanied by the significant inhibition of bone resorption resulting in increased bone strength and improved bone quality. Moreover, the osteogenic compound, forskolin present in high amount in CFE likely contributed to its observed positive skeletal effect.

We used femur osteotomy model as it is suitable for rapid quantitative assessment of bone regeneration due to osteoblastic action *in vivo*. Our dose determination study in this model found a 25 mg/kg oral dose, which is half the adult human equivalent dose to be effective in bone regeneration. Osteogenic efficacy of CFE at lower dose is advantageous as it reduces the possibility of adverse hepatic effects reported in some preclinical studies (8, 35, 36). Postmenopausal osteoporosis is a chronic disease that requires life-long therapeutic intervention or preventive measures. Using 25 mg/kg CFE, we studied the skeletal effects of CFE in OVX rats for three months which is comparable to 9 human years (37).

In growing animals, modeling is the dominant event in bone formation particularly in the cortical shafts of long bones (38). Evaluation of pMS/BS, pMAR and pBFR by dynamic histology at diaphysis in growing rats showed significant increase over the control suggesting enhanced osteoblast activity giving rise to the modeling-directed apposition of periosteal bone. Increased modeling-directed apposition may have contributed to increased bone width as evidenced from increased cortical thickness (Ct.Th) and larger cross-sectional bone area (B.Ar) in the CFE group. In addition, bones with greater cortical thickness will require more strength in bending, and accordingly we observed that femurs of CFE treated rats required greater energy in breaking in three-point bending test, suggesting functional bone accrual. Because peak bone mass achievement have direct consequence on the incidence of fracture risk in old age, we surmise that CFE supplementation by adolescent girls and women till the fourth decade of life before menopause would contribute to maximizing peak bone mass and thereby protect them from the development of osteoporosis and fragility fracture after menopause.

In an osteopenic model of rat induced by bilateral OVX, there is a simultaneous decrease in bone formation and increase in bone resorption (30). In the current study, CFE treatment in OVX rats maintained trabecular bones of both axial and appendicular skeleton. Trabecular bones are readily lost under the estrogen deficient condition leading to compression fracture of spine. We observed that CFE by conserving the trabecular bones afforded resistance against compression collapse of L5 by increasing stiffness. Our *ex vivo* data showed that the OVX-induced loss of mineralizing ability by the stromal cells was maintained by CFE treatment likely by expanding the pool of osteoblast precursor cells that were subsequently recruited to the remodeling site. Expansion of the osteoblastic pool appeared to have increased MAR which is dependent on the number of functional osteoblasts within the basic multicellular unit (BMU) at the remodeling site. Increase in osteoblast precursors and their differentiation in the bone marrow of CFE-treated rats together appeared to enhance the surface-referent bone formation leading to an increase in stiffness.

One of the limitations of mechanical strength testing *ex vivo* is that it is impacted by the size and shape of the bones. We therefore next studied the lamellar-level bone mechanical properties by nanoindentation to assess the mechanical environment to which bone cells are exposed, and subsequently coordinate the adaptation to loads experienced at the whole bone level. The elastic modulus, representing elastic deformation was reduced in the OVX group but was comparable between the sham and CFE groups. Hardness, representing resistance to plastic deformation was also decreased in the OVX group and was comparable between the sham and the CFE groups. A significant increase in the hardness in the CFE over the OVX group indicated the greater formation of new mineralized bone and corroborated the bone formation-promoting effect of CFE. Indentation modulus, also known as indentation stiffness, indicates lamellar-level stiffness and correlates with calcium content (39). A lower value of indentation modulus in the OVX group compared to the sham control suggests greater deformation, which was maintained to the levels of sham by CFE, and may explain higher stiffness in compression test at L5 of CFE group over the OVX.

The carbonate-to-phosphate ratio depicts carbonate substitution in the mineral lattice, and can alter the apatite crystallinity by substitution to phosphate. Our data showed that OVX rats have higher carbonate-to-phosphate and carbonate-to-amide I ratios, which were attributed to high levels of remodeling and variability in bone material composition. A higher carbonate-to-phosphate ratio in the OVX rats would limit crystal growth thereby reducing BMD. An increased carbonate-to-phosphate ratio may positively correlate with fracture risk and bone aging in both humans and animals. Restoration of carbonate-to-phosphate ratio by CFE in OVX rats suggests mitigation of osteoporotic changes that cause a reduction in bone strength.

We next studied the osteogenic effects of forskolin *in vitro* and *in vivo*. The differentiation promoting effect of forskolin in osteoblast is known for a long time (40) although its *in vivo* effects have not been studied. Forskolin stimulated ALP, cAMP and cGMP in calvarial osteoblasts. *In vivo*, it upregulated osteogenic genes in the calvarium of new born pups. Being an AC activator, forskolin induced cAMP like the osteoanabolic drug, PTH. However, unlike PTH, forskolin increased cGMP. One of the major limitations of PTH is the loss of osteogenic window with time due to activation of RANKL *via* the upregulation of PKA pathway (41). Forskolin is known to activate PKA resulting in osteogenic response in osteoblast cultures (41). However, there are equivocal reports regarding its effect on RANKL, one showed increase in RANKL/OPG ratio and the other showed inhibition (41, 42). We observed that despite upregulation of osteogenic genes, RANKL/OPG ratio was unchanged by forskolin. Moreover, because cGMP levels is inversely related with osteoclast formation (43), and we observed that CFE and forskolin increased intracellular cGMP, which may be attributed to decrease in the osteoclastogenic serum marker CTX-1 in the CFE treated OVX rats compared with the OVX rats treated with vehicle. Increased intracellular cGMP levels by CFE and forskolin suggested their guanylate cyclase stimulatory effect beside AC activation, and requires further studies. The unchanged RANKL/OPG ratio further explained why forskolin being a PKA activator did not increase bone resorption marker, CTX-1, in the CFE treatment, unlike PTH. Because CFE treatment maintained the PINP : CTX-1 ratio at the sham level, which had reduced by half in the OVX group, the osteogenic effect of CFE is expected to continue unabated. Such type of mechanism leading to bone conservation has a distinct advantage over PTH which only stimulates bone formation while resorption continues resulting in the loss of its effect over time.

## Conclusions

Our study demonstrated that at a half of human equivalent dose, CFE acts as a dual agent by stimulating bone formation and inhibiting bone resorption in rats, and consequently improves bone mass, strength, and quality. These attributes could potentially afford significant protection from fragility fracture in postmenopausal women. High concentration of forskolin in CFE contributed to *in vitro* and *in vivo* osteogenic effects as well as anti-resorptive effect *in vivo*. Because CFE is already used by humans in the form of a nutraceutical for weight management, our findings in the preclinical models demonstrating significant salutary effects in bone may stimulate conducting clinical studies in postmenopausal osteoporosis.

## Data availability statement

The original contributions presented in the study are included in the article/**Supplementary Material**. Further inquiries can be directed to the corresponding authors.

## Ethics statement

The animal study was reviewed and approved by Institutional Animal Ethics Committee (Registration no.:34/GO/ReBiBt-S/Re-L/99 CPCSEA) (IAEC/2021/16/Renew-0/Dated-04/01/2021).

## Author contributions

NC, LH, and CK conceptualized the idea of manuscript. CK performed major experiments. KP, SS, and SR helped in the osteotomy and OVX surgery. NC and CK wrote and evaluated the manuscript. µCT of bones and calcein labeling assessment in osteotomy study was conducted by CK and VS. CK and AS performed μCT, calcein labeling assessment, bone strength testing, and bone histomorphometry. OVX study was conducted by CK and SB, and the analysis related to trabecular bone parameters, bone strength test experiment, and body composition analysis were conducted by CK. Bone dynamic histomorphometry, *ex-vivo* mineralization, and ELISA were performed and analyzed by CK. qPCR was performed by CK and SrS. AG and LH did the extract preparation and forskolin analysis in the extract. SK and NK performed nanoindentation and FTIR experiments. AP, SPS, and KS performed the relevant phytochemical analyses. All authors contributed to the article and approved the submitted version.

## Supplementary material

Supplementary Figure 1 Qualitative and quantitative analysis of CFE. **(A)** Chromatogram of the acetonitrile at 220nm. **(B)** Chromatogram of forskolin standard at 220nm. **(C)** Chromatogram of the CFE at 220nm. **(D)** Quantification of forskolin and isoforskolin in CFE.

Supplementary Figure 2 CFE decreased fat mass in osteopenic rats. **(A)** Body mass, **(B)** normalized fat mass, and **(C)** lean mass were measured. For parameters shown in **(B, C)**, Echo-MRI was used. All data are expressed as mean ± SEM (n = 6 rats per group); *p <.05, **p <.01, and ***p<0.001 vs. sham.
