# Supplementary figures and images for: A standardized extract of Coleus forskohlii root protects rats from ovariectomy-induced loss of bone mass and strength, and impaired bone material by osteogenic and anti-resorptive mechanisms
Source: Front Endocrinol (Lausanne). 2023 Feb 28;14:1130003. doi: 10.3389/fendo.2023.1130003 (PMC10011618; doi:10.3389/fendo.2023.1130003)

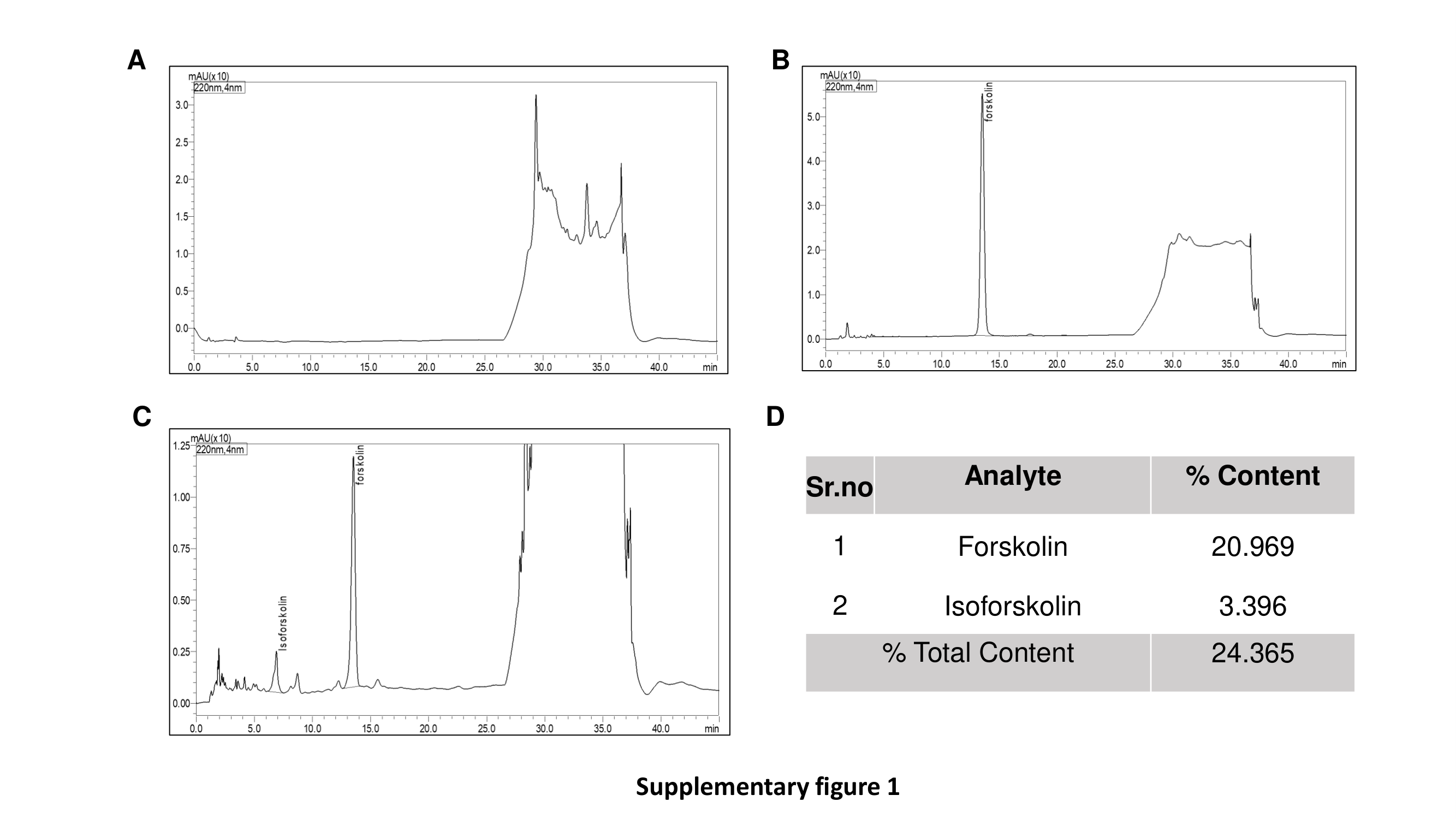

Supplement: Supplementary Figure 1 — Qualitative and quantitative analysis of CFE. (A) Chromatogram of the acetonitrile at 220nm. (B) Chromatogram of forskolin standard at 220nm. (C) Chromatogram of the CFE at 220nm. (D) Quantification of forskolin and isoforskolin in CFE. [file Image_1.tiff]

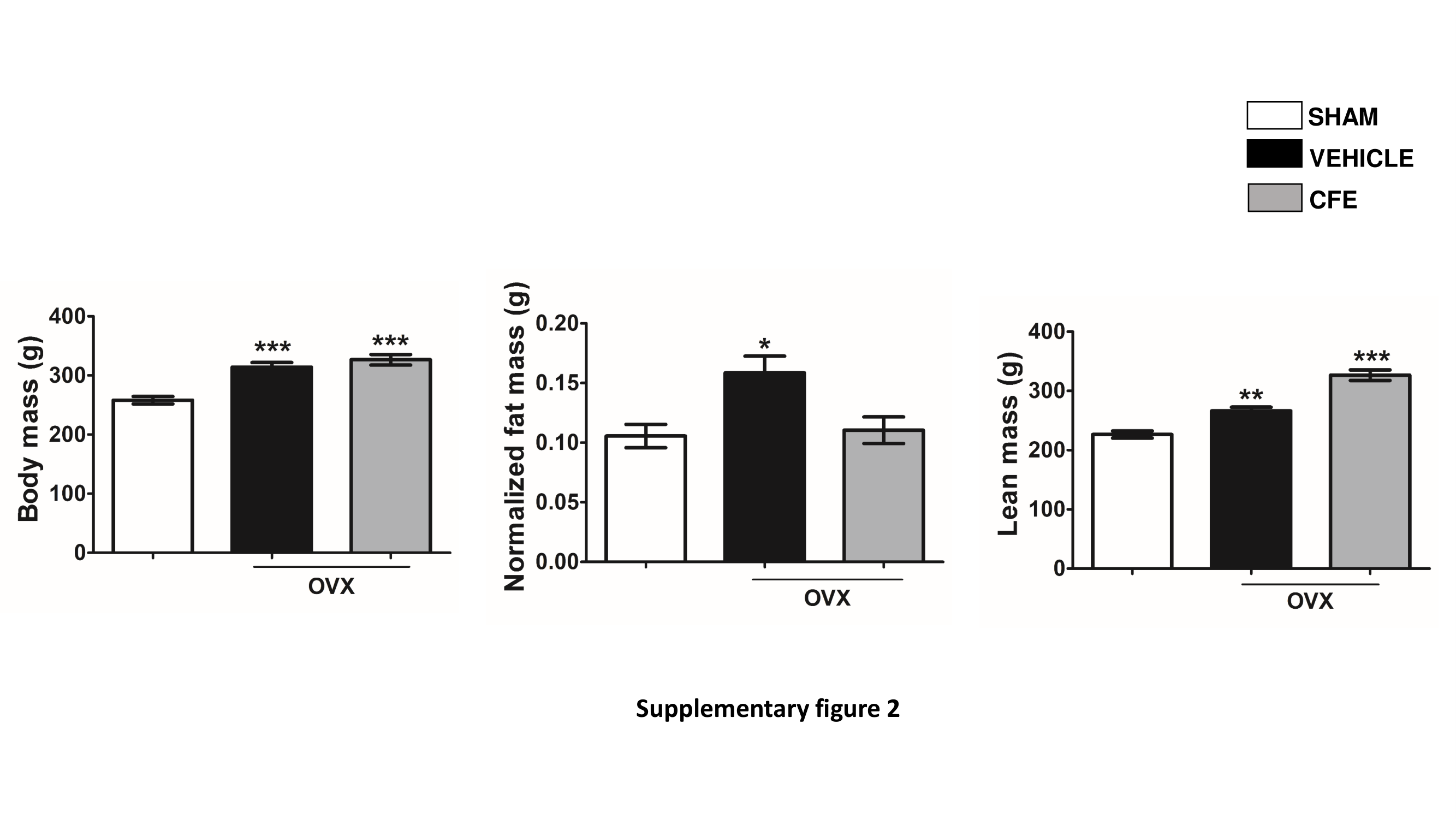

Supplement: Supplementary Figure 2 — CFE decreased fat mass in osteopenic rats. (A) Body mass, (B) normalized fat mass, and (C) lean mass were measured. For parameters shown in (B, C), Echo-MRI was used. All data are expressed as mean ± SEM (n = 6 rats per group); *p <.05, **p <.01, and ***p<0.001 vs. sham. [file Image_2.tiff]
